# Suitability of electroencephalography in brain death determination: a monocentric, 10-year retrospective, observational investigation of 428 cases

**DOI:** 10.1007/s10072-022-06547-1

**Published:** 2022-12-12

**Authors:** Simone Rossi, Gionathan Mazza, Massimiliano Del Testa, Alessandro Giannotta, Sabina Bartalini, Elisa Testani, Laura Savelli, Mario Gabbrielli, Giampaolo Vatti, Sabino Scolletta

**Affiliations:** 1 Department of Medicine, Surgery and Neuroscience, Neurology and Clinical Neurophysiology Section, Siena Brain Investigation and Neuromodulation (Si-BIN) Lab., University of Siena, Policlinico Le Scotte, Viale Bracci, 53100 Siena, Italy; 2 Department of Medicine, Surgery and Neuroscience, Unit of Anaesthesia and Intensive Care Medicine, University Hospital of Siena, Siena, Italy; 3 Department of Molecular and Developmental Medicine, Section of Forensic Medicine, University of Siena, Siena, Italy

**Keywords:** EEG, Brain death, Transplantation

## Abstract

**Background:**

We aimed to verify the usefulness of electroencephalographic (EEG) activity recording (that is mandatory according to the Italian law), in addition to two clinical evaluations spaced 6 h, among the procedures of brain death determination (BDD) in adult individuals.

**Methods:**

The study is a monocentric, retrospective analysis of all BDDs performed in the last 10 years at Policlinico Le Scotte in Siena (Italy).

**Results:**

Of the 428 cases revised (mean age 67.6 ± 15.03 years; range 24–92 years), 225 were males and 203 females. In total, 212 out of 428 patients (49.5%) were donors. None of the BDD procedures were interrupted due to the reappearance of EEG activity (neither for clinical reasons) at any sampling time, with the exception of one case that was considered a false negative at critical reinspection of the EEG. In 6/428 cases (1.4%), a cardiac arrest occurred during the 6 h between the first and second evaluation, thus missing the opportunity to take organs from these patients because the BDD procedure was not completed.

**Conclusions:**

Once the initial clinical examination before convening the BDD Commission has ascertained the absence of brainstem reflexes and of spontaneous breathing, and these clinical findings are supported by a flat EEG recording, the repetition of a 30-min EEG twice over a 6 h period seems not to add additional useful information to clinical findings. Current data, if confirmed in other centers and possibly in prospective studies, may help to promote a scientific and bioethical debate in Italy, as well as in other countries where the EEG is still mandatory, for eventually updating the procedures of BDD.

## Introduction

In 1967, the South African heart surgeon Christian Barnard performed the first organ transplant from a human donor to a human cardiopathic recipient. It was necessary to obtain a still-beating and healthy heart from a donor (a patient with severe post-traumatic brain injury under assistive ventilation) who had to be clinically dead. However, there was no legislation at that time on this issue, and the surgeon himself assumed all ethical responsibilities to pick up the still-beating heart of the donor 3 min after his own decision to stop his ventilatory support [1]. Likely, the donor was in the state of coma depassè, a condition previously described in the fifties as a state beyond the coma, when the patient is not able to breathe, has no reflexes to external stimuli, no motility and no consciousness [2], without overt chance of recovery.

Due to the novel emerging ethical issue and increased attention on the topic, in the following year, a committee of experts was formed at Harvard University to formally define the concept of brain death. They produced a document in which the irreversibility of the coma had to include (1) absence of receptivity and responsivity, (2) absence of spontaneous movements and breath, (3) absence of reflexes, and (4) a flat EEG recording as a confirmation of the clinical diagnosis [3]. About 30 years later, the American Academy of Neurology (AAN) basically confirmed the three necessary conditions (irreversible coma, absence of all brainstem reflexes, and apnea) to define “whole brain death” [4], while in other countries such as Canada and UK, the brain death corresponds to “brainstem death,” as defined by the absence of brainstem reflexes and apnea, thus giving more relevance to vital functions rather than to neocortical activity [5].

The EEG, which reflects thalamus-cortical rather than brainstem oscillatory activity [6], is generally considered among ancillary tests to support clinical examination; sometimes, also another neurophysiological testing, such as brainstem auditory evoked potentials or somatosensory evoked potentials, are considered, as well as other testing addressing cerebral perfusion [7]: indeed, also the absence of cerebral blood flow, including the brainstem, is considered an evidence of brain death, due to the strict correlations between cerebral perfusion and electrical cortical activity [8] (see Discussion Section).

The specific procedures for brain death determination (BDD) (i.e., mandatory observation period(s), number of required examinations, qualifications of medical personnel, use of ancillary testing) largely vary from country to country around the world. In Italy, the current law (number 578 dated December 29, 1993, and following Decreti Ministeriali dated August 22, 1994, and April 11, 2008, and published in the Gazzetta Ufficiale on June 12, 2008) requires two 30-min EEG recordings spaced 6 h as an integral, not ancillary, part in the identification process of an individual’s brain death, as in about a half of the other European countries [9]. The Commission for Death Determination, which includes one anesthesiologist-intensivist, one neurologist expert in EEG, and one forensic doctor, is convened after having verified the loss of brainstem reflexes and the absence of oscillatory brain activity of the individual (or of the cerebral perfusion if the EEG cannot be performed for some reason), generally an in-patient in the intensive care unit.

The primary aims of this study were (i) to verify the real additive value of the second EEG sampling after having ascertained the absence of EEG activity for 30 min 6 h before, at the beginning of the BDD; (ii) to verify also the usefulness of the first EEG recording of the BDD after having already verified the absence of EEG activity, brainstem reflexes and of spontaneous breathing before starting the procedure itself. To accomplish these goals, we retrospectively evaluated all the reports of the BDD Commission collected at our university hospital over the last 10 years, in the period 2012–2021.

## Materials and methods

A retrospective analysis of all procedures of BDD carried out at our university hospital from January 1, 2012, when digital storage of EEG recordings became available, to December 31, 2021, has been performed. The study has been approved by the Local Ethic Committee under the code CAM-2022.

All reports written by the death commission board (the Italian acronym CAM, which means Commissione di Accertamento Morte) concerning adults in this period have been reviewed. The number of interrupted brain death procedures at the first or the second sampling time was recorded, and motivations were collected. In case the interruption of the procedure was due to the presence of EEG activity, digital records were independently reexamined by two neurologists who are experts in EEG, included in the local commission for BDD, and who critically discussed those EEG findings.

## Results

**Fig. 1:**
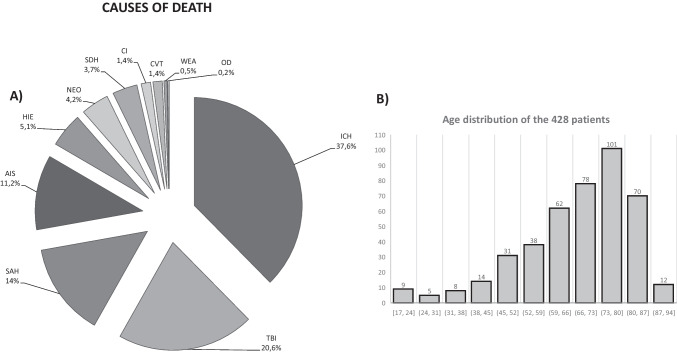
**A** Causes of death in the 428 patients. Legend: ICH, spontaneous hemorrhage; TBI, traumatic brain injury; SAH, subarachnoid hemorrhage; AIS, acute ischemic stroke; HIE, hypoxia; NEO, neoplasia, tumor or metastasis; SDH, subdural hematoma; CI, meningitis or abscess; CVT, cerebral venous thrombosis; WEA, weapon; OD, overdose. **B** Age distribution of the 428 patients. Most of the patients were over 59 years of age (*n* = 285, corresponding to 66.6% of the population)

As shown in Fig. 1(A), 428 cases were collected (225 males and 203 females; mean age: 67.6 ± 15.03 years; range: 17–94 years). In total, 212 out of 428 (49.5%) patients were donors.

The prevalent cause of death was spontaneous hemorrhage (161), followed by traumatic brain injury (88), subarachnoid hemorrhage (60), ischemic stroke (48), and others (see Fig. 1A for the complete picture, with percentages).

Only in one case out of 428 (0.23%), there was an interruption of the BDD due to the description of a “doubtful presence of brain activity” in the first EEG recording; in this case, the commission was reconvened after 6 h and the procedure was successfully concluded. A subsequent critical reinspection of the EEG recording, performed independently by two neurologists, agreed that the doubtful cerebral activity was artifactual.

Irrespective of the patient’s age (see Fig. 1B for age distribution), no procedures of BDD were interrupted for the same motivation at the second EEG sampling after 6 h. No procedures were interrupted, neither at the first nor at the second sampling time after 6 h, for other clinical reasons such as the reappearance of brainstem reflexes or recovery of spontaneous breathing.

In six cases out of 428 (1.4%), a cardiac arrest occurred during the 6 h between the first and second commission evaluation, thus losing the opportunity to pick up organs from these patients because the BDD procedure was not completed.

## Discussion

The main finding of the current study is that the second EEG performed after 6 h of observation seems not to provide additional information on the clinical state beyond those gathered by objective neurological examination and demonstration of the absence of spontaneous breathing; indeed, in none of the 428 cases a reappearance of EEG activity was observed at this sampling time.

A similar consideration could be put forward also regarding the first EEG recording coupled to the clinical evaluation: in our sample of 428 cases, there was only one case with suspected EEG activity of cortical origin (not confirmed after the critical reinspection), hence no procedures of BDD were interrupted due to a true reappearance of oscillatory cortical activity at the first sampling time.

Therefore, the current study suggests that once the initial clinical examination before convening the commission for BDD has ascertained the absence of brainstem reflexes and of spontaneous breathing, and these clinical findings are supported by a silent EEG recording for 15–20 min, the repetition of a 30-min EEG two times spaced 6 h does not seem to provide additive useful information to those derived by the clinical observation only in this time period.

This is not physiologically surprising, taking into consideration the close relationships between the EEG activity and the reduction of cerebral perfusion: reversible oscillatory changes appear when the CBF decreases below 25 mL/100 g/min [10] and the cortical activity is totally suppressed when CBF reduces below 12–16/mL/100 g/min [11]. A few seconds of the absence of perfusion are sufficient to determine neural cell death and loss of brain functions due to irreversible membrane failure in ion transportation [12]. In other words, it is physiologically unlikely that either brainstem reflexes, spontaneous breathing, or cortical oscillatory activity could reappear after about 1 h from the initial clinical and neurophysiological evaluation that determines the convocation of the commission for BDD: indeed, this is the average time generally elapsing from the moment of the decision to proceed with death determination and the formal beginning of the required clinical and neurophysiological procedures according to the current Italian law. Moreover, there is to consider that prolonging the period of observation beyond the first evaluation may result in the loss of organs available for transplantation: this occurred in 1.4% of our sample of cases, that died from a cardiac arrest despite ventilation support.

Thus, current data strongly agree with the suggested practice parameters of the American Academy of Neurology that does not recommend the use of EEG (as well as other ancillary testing) beyond noting the presence of apnea and the absence of brainstem reflexes, with the only exception of conditions that in adults prevent the execution of a reliable clinical/neurological examination, as high blood levels of sedative drugs or severe hypothermia, craniofacial or cervical spinal cord injuries, or severe metabolic derangements [4].

In the current study, no false negative EEG findings emerged (i.e., when the patient meets the clinical criteria of brain death and the EEG suggests residual brain function) [13], but the sensitivity of EEG in detecting the so-called electrocerebral inactivity (ECI) [14] in the presence of clinically evident brain death has been described to vary from 53 to 94.6% (see [7]) in different case series, especially concerning posterior fossa lesions [15] or elevated levels of central nervous system acting drugs [16]. Other factors potentially affecting EEG interpretation during BDD could be represented by environmental artifacts of the ICU and by poor inter-rater reliability [17], although this did not occur in our sample of patients.

The current study has several limitations that should be taken into account due to the ethical implications of such a delicate issue and the possible clinical and organizational consequences: first, it is a monocentric and retrospective investigation. Therefore, results will need to be confirmed in other centers with similar studies, and even prospectively, possibly on a multicentric basis. Second, the mean age of the patients is relatively high (67.6 ± 15.03 years), with most of the population more than 59 years of age (Fig. 1B): in younger subjects, for example, brain resistance (including electrical activity) to insults/lesions could be higher, although this was not the case in the current sample. Moreover, and crucially, we did not include pediatric patients in the studied population, so no suggestions can be put forward regarding procedures of brain death determination in these particular patients.

Neither it is our intention to capitalize on the results of this single-center and retrospective study for claim changes in the current Italian law for brain death determination in adult patients. However, if the results of the current study will be replicated in other national and international centers so that a critical mass of data will be achieved, they would be collectively taken into account to promote a scientific and bioethical discussion on the possibility of revising the timing of death assessment procedures in our country. Following this reasoning, one possibility could be maintaining the EEG (or the investigation of the cerebral perfusion when EEG is not feasible) as mandatory to support the initial decision of going further with the formal activation of the commission for BDD once the absence of brainstem reflexes and spontaneous breathing, coupled with a flat EEG recording, is detected in a patient. Then, once the procedure is formally activated, the clinical evaluation could be supported by the EEG (or another ancillary testing) only in those cases in which brainstem reflexes and/or apnea testing could result not reliable (i.e., the conditions indicated by the AAN) [4].

This would help to optimize the allocation of human resources (medical, technical, nursing, and administrative staff) in the clinical daily activities and will prevent potential loss of organs for transplantation, meanwhile aligning Italy, as well other European countries in which the EEG is still an integral part of BDD procedures, to currently operative guidelines of the AAN and UK, in the frame of a full scientific and ethical respect of the patient’s end of life.
